# The complete mitochondrial genome of the freshwater fish *Traccatichthys pulcher* (Cypriniformes: Nemacheilidae) from China

**DOI:** 10.1080/23802359.2022.2104669

**Published:** 2022-07-29

**Authors:** Xiao Jiang Chen, Lin Song, Wen Zhao Liu

**Affiliations:** a College of Fishery Science and Technology, Jiangsu Agri-animal Husbandry Vocational College, Taizhou City, P.R. China; b College of Fisheries and Life Science, Dalian Ocean University, Dalian City, P.R. China

**Keywords:** Mitochondrial genome, *Traccatichthys pulcher;* Phylogeny, Nemacheilidae

## Abstract

This study aimed to sequence and annotate the complete mitochondrial DNA genome sequence of *Traccatichthys pulcher*. The mitochondrial genome comprised 16,583 bp, harboring 13 protein-coding genes (PCGs), 22 tRNA genes, two rRNA genes, and a control region. The whole genome contained T (25.8%), C (26.9%), A (31.4%), and G (15.9%), showing an obvious AT bias (57.2%). Based on the concatenated protein sequences of 13 PCGs, a phylogenetic tree was reconstructed by the maximum likelihood method, and the topology revealed the monophyly of *Traccatichthys*, and the gathering of *T. pulcher* and *M. pulcher*. The mitochondrial DNA of *T. pulcher* (MZ853162.1) and *M. pulcher* (NC_031581.1) were aligned by the BLAST 2 sequences tool, which showed 97% similarity.

*Traccatichthys pulcher* (Nichols & Pope, 1927) (Cypriniformes: Nemacheilidae) is of great ornamental value (Zheng et al. 2016), and often confused with *Traccatichthys taeniatus* (Pellegrin & chevey, 1936). The typical features that distinguish *T. pulcher* from morphology perspective are dorsal fin rays 3-10-11; anal fin rays 2-5; pectoral fins 1-9-13; ventral fin rays 1-7; gill harrow 11-12; short but high caudal fin; cone-shaped head; three pairs of barbels; body covered by small round scales, no scale on the cheeks; green gray in the body, yellowish stomach; a prominent black spot in the middle of the origin of caudal fin; the distinct black submarginal streaks on dorsal and caudal fins (Pan et al. 1990). Nested-clade phylogeographic analysis based on nucleotide sequences of the mtDNA cytochrome *b* by Qiu et al. (2008) indicated that *T. pulcher* may have originated from the border area of Guangxi and Vietnam where the Dongzhong River, Beilun River and Fangcheng River are located. As the synonym name of *T. pulcher, Micronemacheilus pulcher* is invalid now. However, the organism of *M. pulcher* (NC_031581.1) was indicated to be *T. taeniatus* in GenBank, which confused whether this species should be *T. taeniatus* or *T. pulcher.* The sequence data of *M. pulcher* (KF765806.1) (Chen 2015) was unverified because it was not included in NCBI BLAST databases. This study sequenced the complete mitochondrial DNA genome of *T. pulcher*, and a phylogenetic analysis was accomplished with the available mitogenomes sequences among family Nemacheilidae.

Studies involving laboratory animals follow the ARRIVE guidelines (https://arriveguidelines.org/). The samples of *T. pulcher* (voucher number: ASTIH-21b0616d10) were collected from Beijiang River in Shaoguan City (24.7418 N, 113.3758 E), Guangdong Province of China by Lin Song in Jun 2021, and the samples were identified by morphological identification (Pan et al. 1990). The specimen were preserved in 95% ethanol and deposited at Aquatic Science and Technology Institution Herbarium of Jiangsu Agri-animal Husbandry Vocational College (https://www.jsahvc.edu.cn/, XJ Chen, 2007020030@jsahvc.edu.cn). Total genomic DNA was extracted from the muscle of *T. pulcher* using the Tguide cell/tissue genomic DNA Extraction Kit (Tiangen, Beijing, China) and stored in a deep freezer at −80 °C. The extracted DNA was subjected to sample quality control, DNA library was subsequently constructed and amplified by PCR, followed by size selection and library quality check, and finally library pooling and sequencing were carried out on the Illumina Hiseq platform 2500 (Genesky Biotechnologies Inc. Shanghai, China). The next-generation sequencing raw data (3.18 GB) were assembled using MetaSPAdes 3.13.0 (Nurk et al. 2017; Yang et al. 2021), and the assembled mitochondrial genome sequences were annotated by MitoMaker 1.14 (Bernt et al. 2013) based on the reference sequence of *Schistura incerta* (MK361215), and the analyses were conducted using MetaSPAdes, MitoMaker with default parameters. The phylogenetic tree was based on MEGA − X software (Kumar et al. 2018).

The complete mitochondrial genome of *T. pulcher* comprised 16,583 bp, harboring 13 protein-coding genes (PCGs), 22 tRNA genes, two rRNA genes, and a control region (D-loop). The whole genome contained 25.8% T, 26.9% C, 31.4% A, and 15.9% G, showing an obvious AT bias (57.2%). The length of the 13 protein-coding genes ranged from 168 bp (*ATP8*) to 1,839 bp (*ND5*). Of the 13 PCGs, only the *CO I* gene started with GTG, the remaining 12 PCGs started with ATG as the initiation codon. As for stop codons, seven PCGs (*ND1*, *CO I*, *ATP8*, *ATP6*, *ND4L*, *ND5*, and *ND6*) used TAA, two genes (*ND2* and *ND3*) used TAG. Four PCGs (*CO II*, *CO III*, *ND4*, and *Cyt b*) ended with an incomplete stop codon (T−−, TA−, TA−, and T−−). *T. pulcher* contains 22 tRNAs with lengths ranging from 68 bp to 75 bp. In rRNAs, the shortest was *12S rRNA* (954 bp) and the longest was *16S rRNA* (1,636 bp).

To confirm the phylogeny of *T. pulcher*, the whole mitochondrial genomes of 17 fish species from 8 genera (*Homatula*, *Schistura*, *Traccatichthys*, *Barbatula*, *Triplophysa*, *Claea*, *Tarimichthys*) were selected. Based on the concatenated aminoacids sequence of 13 PCGs, the phylogenetic tree was reconstructed using the maximum likelihood method by MEGA X software, and model (mtREV + F + G + I) with the lowest BIC scores (Bayesian Information Criterion) was considered to describe the substitution pattern the best (Jones et al. 1992), with a bootstrap of 1000 replicates. The phylogenetic analysis showed that *S. longa*, *S. reticulofasciata*, and *S. corica* gathered together, and they formed a sister group with the clade (*T. pulcher* and *M. pulcher*) (Figure 1). The complete mitochondrial DNA sequences and concatenated nucleotides of 13 PCGs of *T. pulcher* (MZ853162.1) and *M. pulcher* (NC_031581.1) were aligned respectively using BLAST 2 sequences tool, which showed 97% similarity. (http://www.kinase.com/blast/wblast2.html), it is speculated that the two kinds of fish may be different species. Species name with GenBank accession number NC_031581.1 may be *T. taeniatus*, which needs more data for verification. The fundamental genetic data of *T. pulcher* in this study would be beneficial for further studies on population genetics and the evolution of the family Nemacheilidae.

**Figure 1.**
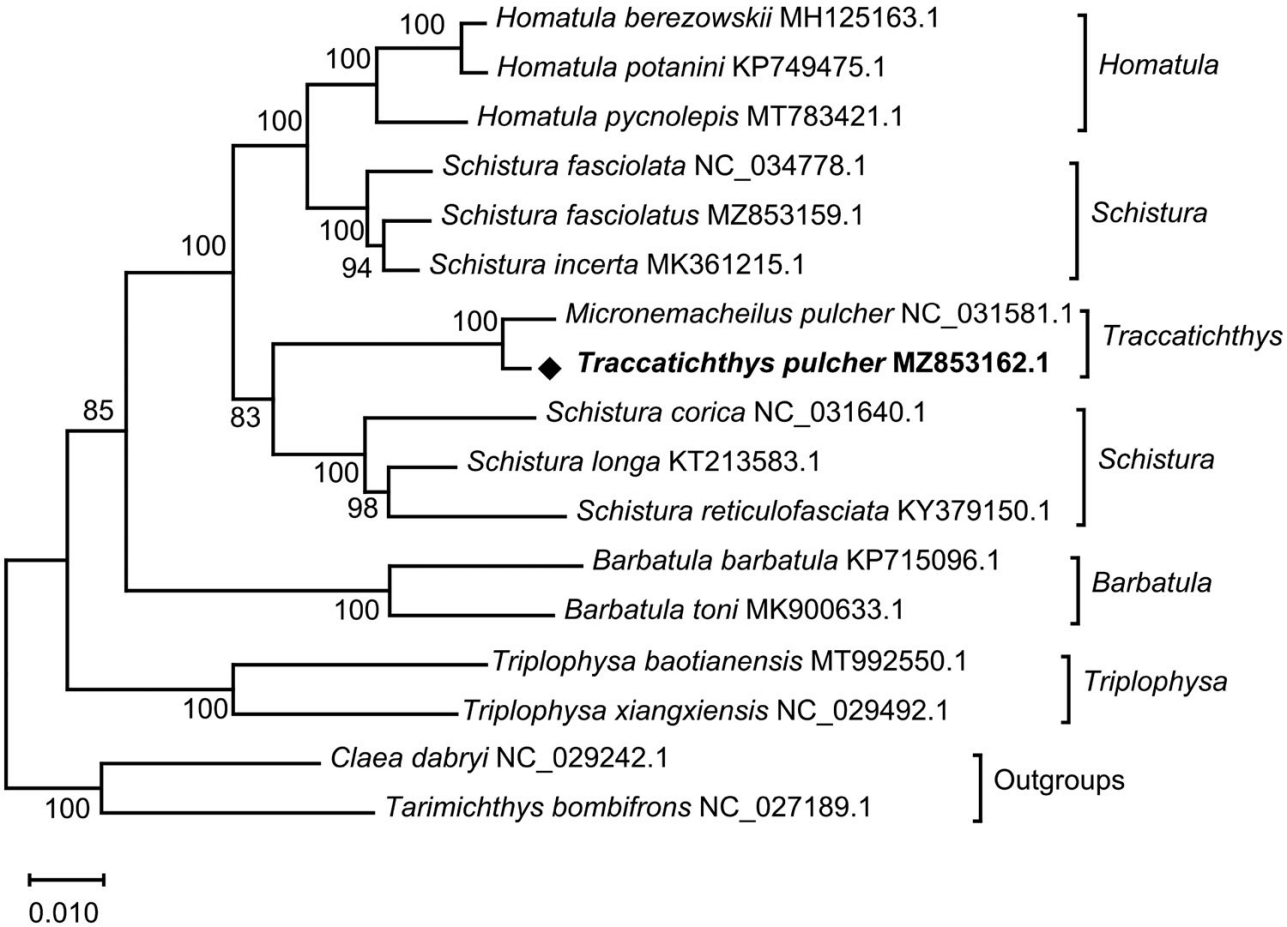
A phylogenetic tree was constructed for the genera *Homatula, Schistura, Traccatichthys, Barbatula, Triplophysa, Claea,* and *Tarimichthys*, with outgroup species, using the Maximum-likelihood (ML) method based on the connected protein sequences of 13 PCGs, with a bootstrap of 1000 replicates. GenBank accession numbers are given with species name, and the numbers at the nodes represent bootstrap values.

## Ethical approval

Experiments were approved by the Ethical Committee for Animal Experiments of Jiangsu Agri-animal Husbandry Vocational College and conducted following the Chinese Association for the Laboratory Animal Sciences and the Institutional Animal Care and Use Committee (IACUC) protocols.

## Author contributions

Conception and design, XJ Chen and L Song; Data curation, WZ Liu and XJ Chen; Analysis and interpretation of the data, L Song and XJ Chen; Funding acquisition, XJ Chen; Writing – original draft, XJ Chen, L Song, and WZ Liu; Writing – review & editing, XJ Chen, L Song, and WZ Liu. All authors agree to be accountable for all aspects of the work in ensuring that questions related to the accuracy or integrity of any part of the work are appropriately investigated and resolved.

## Data Availability

The genome sequence data that support the findings of this study are openly available in NCBI at https://www.ncbi.nlm.nih.gov/nuccore/MZ853162, reference accession number MZ853162. The associated ‘BioProject’, ‘Bio-Sample’, and ‘SRA’ numbers are PRJNA769998, SAMN22190292, and SRR16282473, respectively.
